# Demand for Child Healthcare in Nigeria

**DOI:** 10.5539/gjhs.v4n6p129

**Published:** 2012-09-19

**Authors:** Olanrewaju Olaniyan, Odubunmi Ayoola Sunkanmi

**Affiliations:** 1 Department of Economics, University of Ibadan, Ibadan, Nigeria; 2 Department of Economics, Lagos State University, Ojo, Lagos, Nigeria

**Keywords:** human capital, healthcare, nested logit model, demographic health survey, households

## Abstract

Nigeria with an estimated $350 per capital annually still ranks near the bottom 158 out of 177 countries in the UN Human Capital Development Index in terms of per capita income, with more than half of the population living in poverty. Over the past decade U5MR is estimated to be 201 deaths/1000 lives births, the high rates of child mortality especially the 0-5 years shows the total breakdown of social and economic well-being of the country. This paper examined child health care demand in Nigeria using the Nested Multinomial Logit Model estimation technique. The study used parents’ education as a proxy for child education, while the decision to make a choice of the health facilities was also assumed to be that of the House-Hold head. The study found out that female child has a higher probability of seeking health care facility ahead of their male counterpart. Also, the household head educational level was found to be a determinant of health care seeking behavior of the child. Empirical evidence also revealed that that the probability of seeking healthcare increases with household size and that demand for child health care in Nigeria is non linear in nature. Based on this, the paper recommends the need to show greater commitment to child health care and that government should reduce the problems militating against effective performance of the health sector such as, inefficiency, wasteful use of resources, low quality of service and poor enabling environment.

## Introduction

Health is a fundamental dimension of well-being and a key component of human capital. Conversely, poor health and the inability to cope with episodes of illness can be considered important dimensions of deprivation. Health outcomes are affected by a wide range of factors, pertaining to the individual, social and environmental context. In addition, preventive and curative health services are direct inputs that affect an individual’s health status and ability to cope with ill health (Benefo and Schulz, 1994). However Amponsah (2000) sees child health care as the principal barometer which can be used to assess both social and economic well being of any country. Also nutritional status of U5 Children is considered as a major indicator of a household’s living standard and also determines child survival (Thomas et al, 1990). Nigeria’s estimated population of 120 million in 2002 (projected from the 1991 National Population Census) makes it the largest country in Sub-Saharan Africa and the tenth most populated country worldwide. Nigeria’s population is largely rural, with 63.7 percent of the population living in rural areas. Currently, about 20 percent (24 million) of Nigeria’s total population are under age five (policy project Nigeria, 2002). The huge numbers involved, therefore, required that child healthcare demand be placed in the forefront of the national agenda.

Nigeria is blessed with both human and natural resources, despite this; it is ranked among the 13 poorest countries in the world. The World Bank (2001) reports that majority of Nigerians earns below US $1 a day and this shows high level of poverty in the country. This extreme poverty serves as a limiting factor to access quality health care especially among the vulnerable group (Children) (World Bank, 1999, UNICEF, 1999). Less than half of the population has access to safe water (40% in rural areas) and only 41% have access to adequate sanitation (32% in rural areas). All these facts have negative implication for the survival of the children).

From the foregoing, it is evident that Child survival in Nigeria is threatened by nutritional deficiencies and illnesses, particularly Malaria, Diarrhoeal diseases, acute respiratory infections (ARI), and Vaccine preventable diseases (VPD) which account for the majority of morbidity and mortality in childhood. Other threats include high maternal morbidity and mortality. It is therefore imperative for governments at all levels to formulate policies that can fully develop Nigerian Children and enhance their quality of life.

Realising the importance of Child health care, health policy makers in Nigeria have been directing policies towards solving impediments to health care access among the children in order to improve their health care problems. However, a vivid examination of government health care policies shows that they are titlted towards addressing supply barriers, while the demand side policies have concentrated on improving staff quality, reduction in waiting time, provision of drugs, building of more hospitals and improving the environment of the health care facilities without adequate provision on how people especially the vulnerable ones can have access to these facilities (Ensor and Copper, 2009). Consequently, there have been a wide range of government efforts to address problems facing the child heath care. However, a probe into the literature has shown that supply side of the health care is not enough in addressing health care problems, but it must be mixed with the demand side solutions (Akin et al, 1995). Although, the importance of supply side solutions needs not be relegated to the background, it needs be pointed out that they are not enough in addressing access to health care by patients, especially children in low income countries like Nigeria. It is therefore necessary for policy makers to consider other interventions beyond the supply and reflect on how individuals behave during and the magnitude of the factors affecting their health seeking behavior, especially the children who are socially vulnerable.

This study therefore attempts to find out the factors that will determine child health care and also to know if residency in any of the Geo-political zones in Nigeria will influence health care demand. In this study, health is understood as the probability of seeking different types of care conditional on being ill.

**The rest of this paper has been divided into four sections. Section I reviews relevant literature on child mortality and health care in Nigeria, section II is the research methodology and estimation techniques. Section III presents and discusses the result, while section IV concludes and provides policy recommendations.**

## Section I: Literature Review

Evidence is accumulating on the huge gap between different groups in both developed and developing countries in accessing health care. considerable differences in child survival as a result of income and ethnic groups is well established across Asian, African and South American countries (Ensor and Cooper, 2004). Equally, it has been well documented in the literature that access to health services and the distribution of public subdies favour richer, urban dwellers over generally poorer rural inhabitants (Demery 2000; Makinen 2000, Waters et al., 2000). According to Ensor and Cooper (2004) investments in public sector health care infrastructure have not primarily benefited the most vulnerable in the society, especially children. Considering per capital expenditure on health, statistics has shown that most governments in low income countries spend less than US$4 annually and this has a significant implication for the health care delivery (Jowwet, 1999).

UNICEF (2000) reports that one out of seven Nigerian children die before his or her fifth birthday. NPC (2001) report further confirms that there is thirty percent probability for a baby born in Nigeria to die before attaining five years of age when compared to his or counterpart in developed nations. Statistics show high rates of child mortality in Nigeria and ranked 15^th^ in the world (UNICEF, 2001). It is on record that over one million children die yearly as a result of preventable diseases, this has made Nigeria to be in the fore front of African countries who has not recorded much success in child survival in the last four decades despite their self acclaimed advances in global immunization and oral re-hydration therapy (ORT).

The Nigerian Demographic and Health Survey (2008) shows some improvement in under 5 mortality rate (U5MR) (see table 1), these rates still fall short of the World Summit for Children (WSC) national goals for reducing U5MR (70/80 per 100) by one third by 2010. The Multiple Indicator Cluster Survey (MICS) conducted by unicef in 2008 shows that U5MR was almost 1.5 times higher in urban areas and that almost twice as many children died before their fifth birthday in the North West than in the South west of Nigeria.

**Table 1:** Comparison or rates between 1990 and 2008

YEAR	U5MR	% FALL
1990	191	30%
2008	140	

The study on demand for reproductive Health and child mortality in Nigeria by Adeoti (2009) found an inverse relationship between child immunization and mortality in rural and urban areas, a child who takes all the required immunization has less probability of dying before age five. The level of education of the mother, distance to health care facilities and mother’s age were found to affect demand for child immunization especially in the rural areas (Adeoti, 2009). According to the World Health Organisation (2006), about 60% of all deaths occurring among children in developing countries are as a result of Manultrition. Statistics further shows that about 50.6million under five children are malnourished, while 90% of these children are from developing countries. Growing literature on child mortality and morbidity has reported an inverse relationship between Household Socio-Economic Status (SES) and child mortality in developing countries (Antonovsky and Bernstern 1977, Caldwell 1978, Vanzo 1983, D’souza and Bhuiya 1982, Farah and Preston1982). However to Mosley and Chen (1984) Socio-Economic Status (SES) affects child mortality through Nutritional in-take, thus, there is a positive relationship between Socio-Economic Status (SES) and child nutritional status. Determinants of child health care according to Bhuiya et al.(2010) includes: adequate food intake and proper health care during and after sickness, household resources, attitude of the decision makers towards the children, household size and type of household that the child belongs. Cadwell and Smith (1983) found a positive relationship between mother’s educational background and child health care. Urban poor settlements has also been identified by APHRC (2002) as one of the major factors that pose serious challenges to child health and survival. Majority of the urban residents live in slump settlements that are characterized by poor environmental sanitation and livelihood conditions (Kimawi-Murage and Ngindu, 2007). The macroeconomics and health report emphasized the need to extend essential services and also make structural changes in health services in the poorest countries, especially at the community level in order to overcome most of the important barriers in accessing health care services among the vulnerable groups (Sachs, 2001). Ensor and Cooper (2004) notes that, supply side factor has dominated health care decision and it is only one factor in the decision making processes in the health sector. As they aptly put it” is important that health seekers have knowledge of what providers offer, education about how best to utilize self-and practitioner provided services and cultural norms of trearment”.

## Section II

## Methodology

Following the approach of Lindelow (2002), the empirical analysis of this study is informed by a fundamental economic theory of utility maximization of health. According to this theory, utility of any economic agent *i* 1 depends on the health status of such economic agent and his non-health consumption, represented by *h and x respectively. This is represented as:*


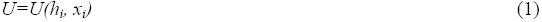


However, Utility will be maximized subject to a production function of the economic agent and his budget constraint. This is presented as:


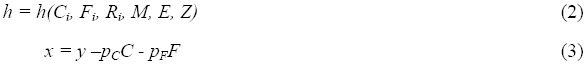


In equation (3), C represents the quantity and quality of health care; F represents other health inputs such as sanitation, food consumption and others; R means individual attributes which include, age and gender; M and E captured both household and community attributes respectively; while Z is a vector which is used to proxy the choice alternatives from available health care providers. *X which represents* nonhealth consumption is exogenous income, y, minus health inputs costs. Further, p_C_C and P_F_F, are direct charges and indirect cost of accessing health care (waiting and travel time). By combining the objective function and the constraints, (that is equations 1, 2 and 3) we form a composite function represented as^1^


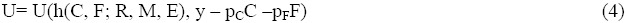


Equation (4) above is a random utility model for polychotomous choice, based on the condition that only the sick will seek for health care and when seeking health care, they are faced with several options represented by J. However, each of these available options differ with respect to their impact on health status of the individual that are sick and also on the direct and indirect cost of such health care (Total cost). If individual opts for choice j, then V^*^_j_ can be defined as the level of indirect utility associated with that alternative:


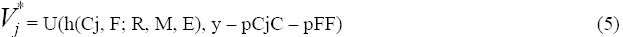


V^*^_j_ contains an error term that shows that the optimization process is not perfect and there could also be inherent measurement error. We therefore define, Vj, as:


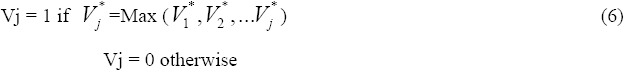


Since we stated earlier that Vj, depends on an individual being sick, this therefore may lead to selection problem. The most common empirical specification of this general framework is the linear model (Lindelow, 2002; Kasirye et al, 2004):


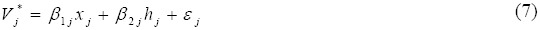


Under this scenario, utility is a function of nonhealth consumption, xj, and health, hj, conditional on receiving care from a health care provider of type j. The constraints are given by


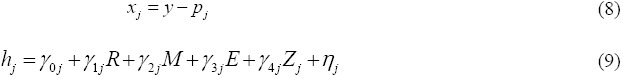


Equation (8) further buttresses our earlier position that non-health consumption is represented by the difference between exogenous income, y, and unit cost of health care (It should be noted here that when an individual is sick he visits or consults the health care provider of his choice) that is provider j, pj.. Equation (9) above expresses Health as a function of individual,(R) Household (M), community(E) and provider/choice(ZJ) attributes. Equation (10) is therefore our estimable equation.


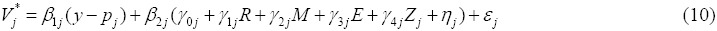


It should be noted however that it is assumed in equation (10) that price elasticity is independent of income^1^. As a result of this Gertler and van der Gaag (1990) and Lindelow (2002)^2^ proposed an empirical specification based on a semiquadratic utility function that is linear in health but quadratic in consumption thus:


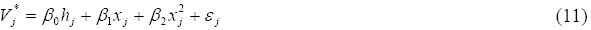


where


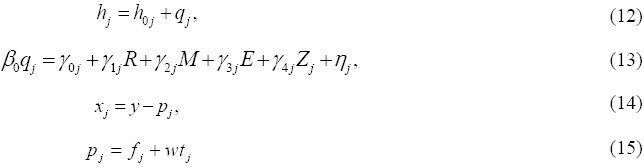


From equation (11) q_j_, is the quality of health that results from the treatment received by an individual as a result of receiving treatment from health care provider j, while equation (12) states that the expected health status as a result of consulting provider j is the addition of the health status as a result of no care (h_0_,) and health care improvement as a result of consulting j provider (q_j_)_._ We therefore specified quality (q_j_) as a parametric function of its earlier stated determinants, where the anticipated health care improvement is brought about by the household production function. The significant variables (13) are individual (R), household (M), community (E) and provider (Z) attributes. Gerter et al (1987) notes that since both marginal utility of an individual’s health and the production of health depend on demographic variables, the effects cannot be identified separately. They therefore proposed a reduced form model where utility is derived from quality. The cost of care from provider *j* is given by the user fee f_j_ and the opportunity cost of time (t_j_) spent seeking care. Combining these equations we get:


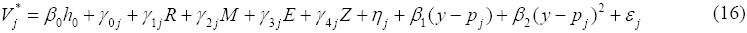


Expanding equation (16):


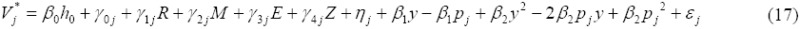


A cursory examination of equation (17^/^) above shows that some observed variables do not change with *j*, because they do not control provider’s choice, as such, we need to drop them, thereby leading to equation (18):


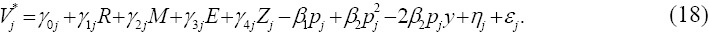


An alternative flexible behavioral model (19) was proposed by Dow (1996a) as a result of the fact that equation (18) as specified allows interaction between price and income:


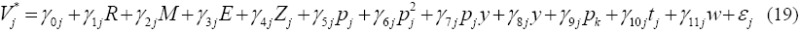


Based on Dow (1996a) observation, there are variations to equation (19) which is in line with recent literature on health care demand (Lindelow, 2002; Karsiye, 2004). One of such variation is that, coefficients on price and price/income variables are allowed to change across health care providers, because we no longer assume additive separability in the utility function. As a result, Dow (1996) proposes the inclusion in the utility function an interaction term between consumption and health improvements. This then means that we can estimate income and price terms with separate alternative-specific coefficients. In addition, Dow introduced changes into the model through the budget period (Lindelow, 2002). On this basis, Dow (1996) specifies residual consumption as:


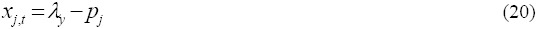


The total cost of care from provider j according to Dow (1996a) is:


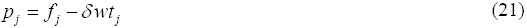


This is based on the fact that the market wage as a result of unemployment and underemployment may over rate the worth of time value. The implication of this is that the coefficient on *wt* can be at variance with that on the user fee. He then suggests different estimation for travel time and wages, which growing literature on health care demand have led credence to (Lindelow, 2002, Ichoku and Leibbrandt, 2003, Karsiye, 2004).

The dependent variable V^*^_j_ in equation (19) represents five possible outcomes or alternatives that are considered in estimation following Lindelow (2002) and Karsirye (2004): (1) no care/self care; (2) traditional medical practitioner; (3) hospital or doctor; (4) health post or nurse; (5) pharmacy, private clinic, or other. The individual attributes include age, gender, education, income, wage rate, and health status (symptoms). Age is a continuous variable, while gender enters as a dummy variable taking the value one for male and zero otherwise. The effect of education is captured through four dummy variables: no education, primary education, secondary education and post-secondary education. It should be noted here that we use education of the parent to represent the child education since healthcare decision will be made by the parents on behalf of the child.

## Description of Variables and A-Priori Expectation

Income is being proxied by total household monthly income proxied by household consumption (Gertler et al, 1987; Gertler and van der Gaag, 1990; Mocan, 2005) or per-capita monthly expenditure (Havemann and van der Berg, 2005), or assets (Akin et al, 1986) or income categories dummies (Akin et al, 1995). The wage rate enters both on its own, and as a determinant of the opportunity cost of time. Wage can be proxied by per capita daily household consumption. Many studies of health care demand also include a measure of health status as an explanatory variable (Akin et al, 1995; Gertler et al, 1990; Lindelow, 2002). Symptoms are included as explanatory variables. This is because there are good theoretical reasons to suspect that symptoms are important determinant of whether and where individuals choose to consult.

On household attributes (E), two are captured through the household variables^1^. First, income is proxied by the number of rooms of the dwelling, and indicator variable for ownership of a television set, radio, vehicle, and bicycle. Further, the household size enters the model as an explanatory variable. Community attributes are also included as explanatory variables. As indicator variables we intend to find out (1) availability and distance of a hospital in the community, (2) availability of transport which is a determinant of health care access. (3) Annual spending on medicines. The quality of care is measured as drug availability and staffing characteristics of facilities. To capture choice attributes in the model, we employed four explanatory variables: (1) price of care from provider j; (2) price squared; (3) a price income interaction term; (4) travel time associated with different forms of care (this is proxied by reported travel time to nearest doctor).

A-priori we expect income, education, wage rate, health status^2^ to have a positive impact on child health care, while price, age, travel time gender will have a negative effect on health care demand.

## Estimation Technique of the Study

This study adopts the use of the nested multinomial logit models (NMLM) estimation techniques in the attempt to determine the demand for child healthcare in Nigeria. This is as a result of inherent problem associated with the multinomial logit model (MLM) which does not assume correlation of error terms. This assumption that error terms are completely independently distributed is what literature referred to as independence of irrelevant alternative (IIA). The need for the study to adopt the nested multinomial logit model is informed by the fact that it employs a sequential choice structure where individual economic agent is assumed to select between differentt classes of utilities at each level. At the same time, correlation between the error terms in the independent classes is assumed to be zero, that is, error terms within the classes are allowed to be non-zero.

## Sources of Data

The data set used for the analysis is the Nigerian Living Standards Survey (NLSS) of 2004 carried out by the National Bureau of Statistics in order to contribute to a better understanding of the current health care system. The choice of the 2004 survey is predicated on the fact that it is the most recent household survey conducted in Nigeria. The Nigerian Survey fits with a number of studies conducted throughout the world, in an effort to have internationally comparable statistics on a number of socio-economic conditions.

The NLSS, like most household surveys, is based on NISH frame. The NISH design is a two-stage design with Enumeration Area’s (EAs) as first stage units and households as second stage units. Ten enumeration areas (EAs) were randomly selected each month and five household were systematically selected from the household listing of each selected EAs. The sampling design for the NLSS was meant to give estimates at National, zonal and state levels. The first stage was a cluster of housing units called Enumeration Area (EA), while the second stage was the housing unit. Population level estimates are made by multiplying the data for each household by two factors, one equal to the inverse of the probability of selecting that household from the total list of households in its EA, and one equal to the inverse of the probability of selecting that EA from the list of EAs in its state. One hundred and twenty (120 EAs) were selected and sensitized in each state while sixty enumeration areas were selected at the Federal Capital Territory (FCT) ten Enumeration Areas (EA) with five housing units were studied per month. This means that fifty housing units were canvassed per month in each state and twenty-five housing units in Abuja. Population level estimates are made by multiplying the data for each household by two factors, one equal to the inverse of the probability of selecting that household from the total list of households in its EA, and one equal to the inverse of the probability of selecting that EA from the list of EAs in its state. The selections were done by treating every unit as the same and using simple random selection. In the survey, data is provided on provider choice, access variables, individual characteristics, household characteristics, facility and community characteristics. The Nigerian study fits with a number of studies conducted throughout the world, in an effort to have internationally comparable statistics on a number of socio-economic conditions. Finally, there is a complete module on health care in the survey questionnaire.

## Section III

## Empirical Results and Interpretation

The result of the nested multinomial logit is presented in Table 4. The first part of the nested multinomial result highlights the predicting form of facility chosen while second part reveals the choice of the individual with respect to seeking health care against no care. The results of both models were obtained by estimating the full-information maximum likelihood nested logit procedure. The sample result (0-5 years) is presented in Table 4.

**Table 3:** Description of variables

	Description of Variable	Mean	Standard deviation
**Facilities Characteristics**			
No Care	The respondent receives no care.	5.66	3.18
Hospital	The respondents sought for care in the hospital	4.22	3.30
Health post	The respondents sought for care from a health post	3.40	3.06
Pharmacist	The respondents sought for care from chemist/pharmacist shop	4.07	3.223
Traditional	The respondents sought for carefrom traditional healthcare facilities	4.785	3.80
Consultation Fees	Amount paid for consultation in Naira	370.75	720.08
Transportation cost	Amount paid on transportation from respondent’s house to the place where health care was sought	162.49	490.71
Consultation Time	Waiting time before being attended to at the facility	4.40	10.85

**Individual Characteristics**			
Male	1 if the respondent is male	4.152	3.40
Age	age in years	40.25	12.22
Age2	Age in years squared divided by 100	16.25	5.11
Mot_no educ	The mother has no formal education	3.09	2.92
Mot_pry_educ	The mother head highest educational attainment is primary education	4.12	3.31
Mot_sec_educ	The mother highest educational attainment is secondary education	5.32	4.21
Mot_psec_educ	The mother highest educational attainment is post secondary education	5.94	4.33
fat_no educ	The father has no formal education	3.21	2.93
fat_pry_educ	The father head highest educational attainment is primary education	3.43	3.02
fat_sec_edua	The father highest educational attainment is secondary education	4.95	3.41
fat_psec_educ	The father highest educational attainment is post secondary education	4.32	3.03

**Household Characteristics**			
Household size	Number of people in the household	6.77	2.98
Log of PC H/h Expenditure	Log of per capita household expenditure	3.358	3.12
Sqd log of PC H/h Exp.	Squared log of per capita household expenditure	0.517	0.123
Monogamous	Married and in a monogamous household	0	0
Polygamous	Married and in a polygamous household	0	0
Divorced/Separated/widowed			
Disease Characteristics			
Days activities stop due to illness	Number of days activities stopped due to illness	4.71	3.18
Injury	Number of days activities stopped due to injury	4.58	3.28
Both injury and illness	Number of days activities stopped due to illness and injury	9.23	3.98
Community Characteristics			
Urban	1 if household residence location is urban	4.614	3.02
North east	1 if household’s location is North east	3.76	3.12
North West	1 if household’s location is North west	3.40	3.14
North Central	1 if household’s location is North Central	4.24	3.02
South East	1 if household’s location is South east	6.722	3.47
South West	1 if household’s location is South west	6.4	3.38
South South	1 if household’s location is South South	6.19	3.23

**Table 4:** Nested logit regression result using traditional as the base category

	0-5 (2)		Male		Female	
**Facilities Characteristics**						
No Care	3.315	(0.000)	-0.064	(0.002)	1.890	(0.011)
Hospital	0.951	(0.000)	1.344	(0.000)	2.981	(0.000)
Health post	1.948	(0.000)	-0.425	(0.0012)	-2.435	(0.000)
Pharmacist	1.277	(0.000)	3.120	(0.005)	1.982	(0.000)
Consultation Fees	0.001	(0.000)	-0.002	(0.000)	0.146	(0.000)
Transportation cost	0.010	(0.000)	0.011	(0.124)	0.010	(0.000)
Consultation Time	0.234	(0.000)	-0.346	(0.000)	1.548	(0.001)

**Individual Characteristics**						
Male	0.001	(0.928)	0.243	(0.234)	1.320	(0.002)
Log of age	-0.176	(0.305)	-0.156	(0.000)	-1.452	(0.005)
Primary education	0.038	(0.735)	1.382	(0.142)	2.456	(0.000)
Secondary education	0.336	(0.023)	0.233	(0.004)	1.432	(0.003)
Post secondary education	0.381	(0.143)	1.423	(0.024)	0.569	(0.010)

**Household Characteristics**						
Household size	-0.008	(0.696)	-0.823	(0.004)	2.235	(0.002)
Log of PC H/h Expenditure	-0.097	(0.919)	-0.217	(0.713)	-1.987	(0.013)
Sqd log of PC H/h Exp.	0.069	(0.602)	0.0046	(0.406)	2.142	(0.322)
Monogamous	0.694	(0.335)	0.197	(0.011)	2.380	(0.014)
Polygamous	0.603	(0.410)	0.346	(0.005)	3.127	(0.120)
Once married	0.403	(0.592)	0.253	(0.023)	2.142	(0.006)

**Disease Characteristics**						
Days activities stop due to illness	0.192	(0.000)	1.980	(0.000)	2.345	(0.006)
Injury	0.034	(0.923)	1.342	(0.020)	2.110	(0.013)
Both injury and illness	-0.773	(0.000)	-0.645	(0.865)	-0.348	(0.629)

**Community Characteristics**						
Urban	0.010	(0.942)	0.398	(0.434)	-2.112	(0.003)
North West	-0.104	(0.442)	1.925	(0.334)	0.658	(0.211)
North Central	0.060	(0.709)	0.968	(0.052)	1.432	(0.221)
South East	-0.215	(0.255)	0.547	(0.071)	-0.347	(0.210)
South West	-0.754	(0.001)	0.986	(0.004)	0.876	(0.003)
South South	-0.655	(0.000)	1.256	(0.000)	0.964	(0.012)
Diagnostics			
LR test for IIA	42.53	(0.000)				
N	12090		6407		5683	
LR chi2	2136.297 0.0000)		1432.89	(0.000)	1654.11 (0.000)	
Log-likelihood	-2823.4726		-2367.86		-1654.4	

*Note: P-values in brackets. Author’s computations using STATA for Windows Version 9.0; underlying data from NLSS, 2004*

As mentioned earlier, the nested logit model was used in order to reduce the problem of independence of irrelevant alternatives (IIA) that results from estimating a non-nested multinomial logit model. An example of IIA in this application is that the log odds of using health care facilities vs. no health care would not be affected by the presence of traditional health care. In order to assess the appropriateness of the nested logit model, a Likelihood Ratio (LR) test was used. The LR test reported at the bottom of Table 4 is a test for the nesting (homoskedasticity) against the null assumption of homoskedasticty). The Chi-Square statistics and associated p-values for each of the four models support the use of the nested logit model.

## Predicting Facility Treatment vs. No Treatment

### Individual Characteristics

In the sample (0-5) result of Table 4, although it was found that females have a higher probability of seeking health care facility ahead of their male counterparts, it was not found significant. This suggests that the gender effect of seeking health care is tilted towards the female’s category, but not pronounced. The result shows that for 0-5 age group there is insignificant likelihood of males seeking health care ahead of females. However, note should be taken that the health care decision for this age group is made by the parent.

The age effect is negative and also significant for this group. Education enters the model in form of dummies with no education as the base category. Children (0-5 years) under consideration were assigned education of the household head. The result shows that primary and secondary education is associated with the probability of seeking health care in this age group. Secondary school education was found sufficient in some other cases. The associated level of seeking health care with increasing level of education is more pronounced for these age groups as household head educational level (primary and secondary school) is a determinant of health care seeking behavior for the child.

### Household Characteristics

With respect to the household characteristics, empirical evidence revealed that the probability of seeking health care increases with household size. For age groups 0-5 the result shows that while demand for health care increases with the number of adult members in the household, increases in the number of children members reduces demand. The proxy for income (per capita household expenditure) was not found to be a significant factor with respect to the probability of seeking health care. The sign was also not consistent with the a-priori expectation of a positive sign. However, the squared of the proxy was found to be a positive determinant of the probability of seeking health care although insignificant. This suggests that demand for child healthcare in Nigeria is non-linear. In addition the result shows that the monogamous nature of a family increases the probability of seeking healthcare.

### Disease Characteristics

The numbers of day’s activities are stopped due to illness of the child increases the probability of seeking healthcare relative to no care. The number of days of sickness of the child was also found to be increasing with these age groups. Household head will demand for health care on behalf of the child as a result of the number of days for which the child has been sick. Having one form of injury or the other did not bring out the probability of seeking health care. However, being sick and injured declines the probability of seeking child health care in Nigeria.

### Community Characteristics

Geographical location entered the model of the demand for child healthcare with a dummy variable urban and rural. Rural location serves as the base category. The specific location that the household is residing was not found to be a significant factor that influences the probability of seeking health care. Household residing in South East, South West and South- South are significantly less likely to visit a healthcare provider compared to their counterparts in North East. By contrast, household residing in the North West and North Central region of the country are more likely to seek health care for providers relative to their North Eastern counterpart.

## Predicting Form of Facility Chosen

### Facilities Characteristics

Thereafter, we turn our attention on what determines the type of facilities that is chosen. In this case, we make use of three facility characteristics namely: consultation fees, transportation cost, and consultation time. The results obtained are quite intuitive. Rather than the expected negative sign for the variables, positive sign were reported with respect to the determinants of child healthcare facilities chosen. Different explanations can be offered to explain this result. It could be because since the child is already sick higher consultation cost, consultation time and transport cost is seen as better solution. The length of period that a child is sick and the suggested solution of health care wherever it may be located, is therefore, a probable explanation for this paradox. This is clear-cut in the Ichoku (2000) study on Nigeria where he posits that the health care facility from which individuals seek health care from depend to a great extent on the level of illness and not on the income or price of the service.

## Section IV

## Summary of Findings and Conclusion

The study shows that female child has a higher probability of seeking healthcare facility ahead of their male counterpart. This suggests that the gender effect is tilted towards the female counterparts and as such there is significant likelihood of female seeking health care ahead of male. However, note should be taken that the healthcare decision of this age group is made by their parents. Again that the household head educational level is a determinant of healthcare seeking behavior for the child. Empirical evidence has also revealed that the probability of seeking health care increases with household size. Further, per-capital household expenditure was not found to be significant with respect to the probability of seeking healthcare. This suggests that demand for child healthcare in Nigeria is non-linear. Number of days activities are stopped due to illness of the child increases the probability of seeking healthcare to no care. The specific location that the household is residing was found not to be a significant factor that influences the probability of seeking child healthcare. Consultation fees, transportation cost and consultation time were found not to be significant in determining facilities choice for the child.

## Conclusion

The magnitude of infant mortality in Nigeria shows that child healthcare demand has not been significantly addressed by the policy makers. Therefore; there is need for stronger commitment to child healthcare. We need to reduce the problems militating against effective performance of the health sector such as; inefficiency, wasteful use of resources, low quality of services, unmotivated workforce and poor enabling environment.

## Footnotes

1 To see this, it suffices to note that the difference in utility between two choices does not depend on income, and hence income does not affect choice.

2 Lindelow (2002) also notes that under the linear model, the marginal rate of substitution of consumption for health is constant, which is inconsistent with health being a normal good.

1 However, other characteristics of the dwelling such as presence of latrine/water closet and water source can not be considered on the grounds that these variables not only proxy for wealth but are also determinants of health status and illness incidence.

2 This is on the basis that a healthy individual will seek lesser health care.
